# A Linear Objective Function-Based Heuristic for Robotic Exploration of Unknown Polygonal Environments

**DOI:** 10.3389/frobt.2018.00019

**Published:** 2018-03-07

**Authors:** Russell Graves, Subhadeep Chakraborty

**Affiliations:** ^1^CoSMoS Laboratory, Mechanical, Aerospace and Biomedical Engineering, University of Tennessee, Knoxville, TN, United States

**Keywords:** multirobot exploration, visibility-based deployment, art gallery problem, environments with holes, next-best-view optimization

## Abstract

This work presents a heuristic for describing the next best view location for an autonomous agent exploring an unknown environment. The approach considers each robot as a point mass with omnidirectional and unrestricted vision of the environment and line-of-sight communication operating in a polygonal environment which may contain holes. The number of robots in the team is always sufficient for full visual coverage of the space. The technique employed falls in the category of distributed visibility-based deployment algorithms which seek to segment the space based on each agent’s field of view with the goal of deploying each agent into the environment to create a visually connected series of agents which fully observe the previously unknown region. The contributions made to this field are a technique for utilizing linear programming methods to determine the solution to the next best observation (NBO) problem as well as a method for calculating multiple NBO points simultaneously. Both contributions are incorporated into an algorithm and deployed in a simulated environment built with MATLAB for testing. The algorithm successfully deployed agents into polygons which may contain holes. The efficiency of the deployment method was compared with random deployment methods to establish a performance metric for the proposed tactic. It was shown that the heuristic presented in this work performs better the other tested strategies.

## 1 Introduction

### 1.1 Motivation

Robots provide solutions for tasks which are too dangerous or too repetitive to be effectively performed by a human. Robotic agents have been employed on a wide scale in applications which allow the agent to be mounted in a stationary fashion and repeat certain operations with little or no change in the series of motions and actions. Single robot systems have been designed to explore unknown environments in order to expand the number of potential applications for autonomous agents. Recently, solutions for exploration of unknown environments by systems of autonomous robots have become a focus in the controls community. Many of the algorithms developed in this field focus on the problem of finding a next best view location (González-Banos and Latombe, 2002). These algorithms use heuristics to determine the best positions to deploy agents in order to complete a map of the environment. This work aims to demonstrate the feasibility of using linear objective functions to describe the next best view problem as an alternative to other available heuristics.

### 1.2 Problem Statement

This work presents a solution to the problem of determining the next best view location for a team of autonomous agents exploring an unknown environment. Each robot considered possesses a set of common traits enabling them to function:

– A unique identifier which describes the agent as unique to its counterparts.
– Laser scanner with no noise and effectively infinite scan range.
– Limited line of sight communications capabilities with no data loss.
– GPS system allowing agent to localize.

These automata are tasked with developing a complete map of an environment which is considered to be an unknown static polygon which may contain holes. Doors or openings in the environment are considered viable paths if they provide an opening wide enough for the robotic agent to pass through.

## 2 Literature Review

### 2.1 Image Segmentation and Feature Extraction

Tests were run with MATLAB wherein a portable network graphics (PNG) image of an environment was imported and converted into an occupancy grid. Agents were deployed onto the map, and it was necessary to design an algorithm capable of extracting the features using a simulated laser rangefinder. This process of extracting and simplifying features from a 2-dimensional image is well explored. Simplistic algorithms such as regular sampling are very quick, but do not consistently yield accurate results (Heckbert and Garland, 1997). Voting methods are also used wherein a number of line segments must agree before a line feature may be extracted (Fernandes and Oliveira, 2008). Decimation wherein arcs are split with chords based on arc to chord distance thresholds to extract the environment edges based on combination of chords (Boxer et al., 1993). One of the most popular algorithms to accomplish curve simplification is the Ramer-Douglass-Peucker algorithm (Heckbert and Garland, 1997). This was chosen due to the method’s low complexity and ability to easily extract features from the noiseless data provided by the simulated stationary agent’s laser.

### 2.2 Mobile Robot Exploration

The problem of deploying agents to cover a known space was first posed by Victor Klee to Vaćlav Chvátal in 1973 (Chvatal, 1975). From this, the first upper bound was established, and the solution was later proved using a 3 coloring technique and expanded in a number of works (O’rourke, 1987; Kröller et al., 2012). The problem of placing or moving agents in a known region has been solved as an NP hard or APX hard problem in the number of vertices (Obermeyer et al., 2011). Heuristic methods for developing trees of agents or Voronoi diagrams are employed to accomplish agent deployment without exact solutions (Cortés, 2008; Schwager et al., 2011). The problem of exploring unfamiliar environments is a logical progression from deploying agents in known spaces. Recently, algorithms have focused on deploying teams of agents which must concatenate a series of environment scans into one cohesive map. This has been approached using occupancy grid and feature-based representations of the known environment as well as simple behavioral models (Cepeda et al., 2012; Aguilera et al., 2015). Algorithms acting on occupancy grid representations employ approximations of whether unexplored cells are free or occupied to estimate the utility of cells (Stachniss, 2009; Costanzo et al., 2012; Potthast and Sukhatme, 2014). Cell-based approaches may deploy agents to frontier cells, cells of high utility, or establish utility gradients or value functions which may allow for the deployment of multiple agents simultaneously (Solanas and Garcia, 2004; Bautin et al., 2012; Andre and Bettstetter, 2016). Other works establish a feature-based representation of the space in order to determine next best viewing position. The deployment strategy explored in Chvátal’s theory deployed agents to the vertices of the environment, and some works utilize this strategy by deploying either to the vertices of the environment or the visible space (Ganguli et al., 2006, 2007; Obermeyer et al., 2011). However, many algorithms leverage the properties of convex star-shaped polygonal regions established at each subsequent viewing location (Ganguli et al., 2007; Obermeyer et al., 2011). The algorithm we present falls into the latter category.

## 3 Technical Approach

This work presents a heuristic for determining the next best viewing location based on a linear program which optimizes the amount of area uncovered with each action taken by an agent. These linear programs may be solved in polynomial time for each automata using the interior point method contained in MATLAB’s legacy code base (Zhang, 1998; Nguyen et al., 2005). In order to format the problem of solving for next best view point as a linear program, both linear objective functions and a set of linear bounds which provide a convex polytope over which the objective function can be minimized.

## 4 Next Best View Heuristic

This work presents three formulations for the linear objective functions and two formulations for the boundaries. The combination of objective function and bounds is determined by algorithm and type of agent. Automata are considered to be in one of the three following states:

- Active and mobile (AM)
- Active and stationary (AS)
- Inactive and stationary (IS)

### 4.1 Universal Algorithms

Regardless of agent type, a set of universal algorithms are employed. These processes are divided into sections including read sensor data, optimize position, deploy additional agents, and concatenate map. These sections were built using MATLAB and tested using PNG images of maps as unknown spaces. We define *α* to be the tuple that defines the 2-dimensional position of an agent *i*. Every robot deployed includes a laser scanner for which a function mimics a 360° scan of the environment by a robotic agent at a position, *α_i_*, in the environment. Post processing of this scan determines map features visible to the agent. The PNG image produces an array structure in MATLAB wherein each cell coordinate may be marked as a logical 1 if occupied or 0 if unoccupied. This entire laser scan process is imitated with the algorithm outlined in Algorithm 1 wherein angular resolution may be decided by the user. For this experiment, a fine resolution was utilized to eliminate the potential for false frontiers. The postprocessing of these scan data tests each ordered pair of laser points, [*x_s_*, *y_s_*], from the set of *n* tuples for any gaps where

(1) $$\sqrt{{\left(x_{s+1}-x_{s}\right)}^{2}-{\left(y_{s+1}-y_{s}\right)}^{2}}>w,\;\text{for}\;s=1,2,\ldots ,n-1,$$

$$\begin{array}{l} x=\text{x coordinate of laser scan point}\\ y=\text{y coordinate of laser scan point}\\ w=\text{width of agent}\left(m\right)\\ n=\text{number of laser scan tuples}\\ s=\text{unique laser scan tuple} \end{array}$$

in which *w* represents the width of the agent. Robot width acts as a threshold defining the minimum free space between walls which an agent may consider as a viable path for exploration. Splitting the ordered list of scan data at these gaps yields a set of *k* clusters and *j* gaps equal to one less than the total number of clusters. Since the divisions between each cluster represent viable areas into which the agent might move to continue exploring the space, the *j* gaps observed by the *i*th agent are defined as frontiers, $F_{j}^{i}$, which represent the boundary between explored and unexplored spaces in the environment 2.

**Algorithm 1:** Read Map Data.

**Input:** Load logical map data
**Input:** Initialize angle *θ* at 0
**While** *θ* ≤ 360 **do**
**repeat**
Initialize *r* = 0 *m*;
Calculate *x* and *y* coordinates;
*x* = *rcos(θ)*, *y* = *rsin(θ)*;
**if** (*x*, *y*) = = 1 **then**
Wall encountered, save pair (*r*, *θ*), and stop;
**else**
*r* = *r* + *s*;
**end**
**until** *stop*;
Increment *θ*;
**end**

The proposed solution leverages properties of star-shaped polygonal environments to provide a bounding set for the resulting linear equations. Isolating the star-shaped region formed by the visible environment features begins by employing a Ramer Doublass Peucker algorithm to produce corners, and the clustering performed prior allows for increased performance of this algorithm (Howard et al., 2002). The technique, outlined in Algorithm 2, is employed to perform an iterative end point fit line extraction on each cluster *C_k_*.

**Algorithm 2:** Iterative End Point Fit.

**Input:** Load sets of laser data points *S* = {(*x*_1_, *y*_1_), (*x*_2_, *y*_2_), …, (*x_n_*, *y_n_*),}
create subset *s*_1_ = *S* **for** *Each set of points, s_j_ do until no new sets are created* **do**
fit line, *l*, between (*x*_1_, *y*_1_) ⊂*s_j_* and (*x_n_*, *y_n_*) ⊂*s_j_*;
find distance, *d* between each point, (*x_i_*, *y_i_*) ⊂*s_j_* and *l* **if** max(*d*) = (*x_m_*, *y_m_*) ⊂*s_j_* > *threshold*
**then**
Split sets at *m* into two new sets and reset numbering;
$s_{j_{a}}=\left\{\left(x_{1},y_{1}\right),\left(x_{2},y_{2}\right),\ldots ,\left(x_{m},y_{m}\right),\right\}$;
$s_{j_{b}}=\left\{\left(x_{\left(m+1\right)},y_{\left(m+1\right)}\right),\left(x_{\left(m+2\right)},y_{\left(m+2\right)}\right),\ldots ,\left(x_{\left(n-\text{m}\right)},y_{\left(n-\text{m}\right)}\right),\right\}$;
$s_{j}=s_{j_{a}}$;
$s_{\left(j+1\right)}=s_{{\text{j}}_{b}}$;
**end**
**end**

This process results in an array of lines defined by their start and end point coordinates.

The end point coordinates indicate the presence of a corner at that location, and this provides the basis for the star-shaped polygon. These values are calculated by every agent at each deployment step.

### 4.2 Active and Mobile

An active and mobile unit is an agent for which the star kernel is a region with area greater than zero inside the subset of the environment observable by the agent. A set of boundary conditions and objective functions are fabricated for these agents to facilitate a transition from the agent’s current position to a more optimal location for exploring unknown areas of the environment.

#### 4.2.1 Objective Function

**Figure 1:**
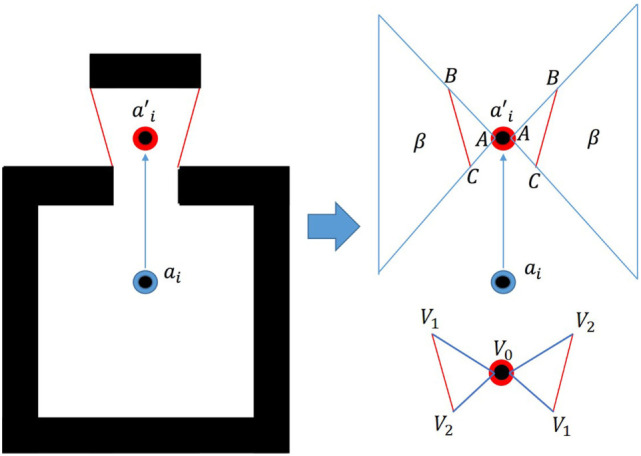
Maximizing area beyond each frontier.

The goal of the algorithm is to develop a linear objective function which may be minimized to yield the next best view. The problem of exploring can be equated to the discovery of the area beyond each frontier displayed in Figure 1.

**Figure 2:**
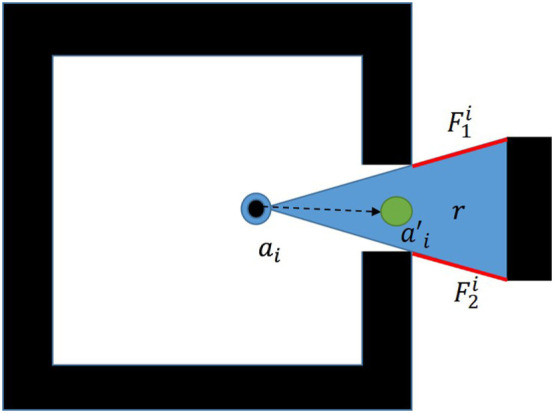
Region in which the vertex ordering is constant.

To maximize the area discovered, *β*, the algorithm attempts to draw the agent to a position, *a_i_* which maximizes the sum of all angles ∠ *BAC* formed with each frontier. The combination of angles is maximized when the area or the length of the side vectors of each Δ *ABC* approach zero. It is well known that the area of a cross products of its two edge vectors, illustrated by equation (2):

(2) $$\begin{array}{ll} \beta \left(\Delta \right) & =\frac{1}{2}|v\times w|=\frac{1}{2}|\left(V_{1}-V_{0}\right)\times \left(V_{2}-V_{0}\right)|\\ \beta \left(\Delta \right) & =\text{Triangle of unexplored area} \end{array}$$

$$\begin{array}{l} v=\text{Triangle edge vector 1}\\ w=\text{Triangle edge vector 2}\\ V=\text{Triangle vertex} \end{array}$$

wherein the vectors *v* and *w* represent the edge vectors of a triangle and the vertices *V*_0_, *V*_1_, and *V*_2_. A 2*d* triangle’s area may be found through a combination of its vertices using equation (3):

(3) $$2\beta \left(\Delta \right)=\left(x_{1}-x_{0}\right)\left(y_{2}-y_{0}\right)-\left(x_{2}-x_{0}\right)\left(y_{1}-y_{0}\right),$$

$$\begin{array}{l} x=\text{Triangle vertex x position}\\ y=\text{Triangle vertex y position} \end{array}$$

in which the vertices are listed as (*x*_0_, *y*_0_), (*x*_1_, *y*_1_), and (*x*_2_, *y*_2_). The signed quantity of this area denotes the orientation of the vertices *V*_1_ and *V*_2_ from *V*_0_. A negative quantity indicates a clockwise orientation of the vertices, and a positive result indicates a counterclockwise order. We take *V*_0_ to be a test position of the *i*th agent, $a_{i}^{\prime }$, and the other vertices as the end points for each *j*th frontier, $F_{j}^{i}$, to get equation (4):

(4) $$\begin{array}{ll} \beta_{j}^{i}\left(\Delta \right)=\frac{1}{2} & \left|\left(F_{j_{x_{1}}}^{i}-a\prime_{i_{x}}\right)\;\left(F_{j_{y_{2}}}^{i}-a\prime_{i_{y}}\right)\;-\;\left(F_{j_{x_{2}}}^{i}-a\prime_{i_{x}}\right)\;\left(F_{j_{y_{1}}}^{i}-a\prime_{i_{y}}\;\right)\right|\\ \beta_{j}^{i}\left(\Delta \right)=\frac{1}{2} & \left|F_{j_{x_{1}}}^{i}F_{j_{y_{2}}}^{i}-F_{j_{x_{2}}}^{i}F_{j_{y_{1}}}^{i}+a\prime_{i_{x}}\;\left(F_{j_{y_{1}}}^{i}-F_{j_{y_{2}}}^{i}\right)\;+a\prime_{i_{y}}\;\left(F_{j_{x_{2}}}-F_{j_{x_{1}}}^{i}\;\right)\right|, \end{array}$$

$$\begin{array}{ll} i & =\text{Robot ID number}\\ j & =\text{Frontier number}\\ a\prime & =\text{Agent position} \end{array}$$

which is the area captured by the triangle formed by the end points of a single frontier and a selected agent deployment position. The agent’s position is always taken to be vertex, *V*_0_, and the ordering of the frontier end points is changed such that the signed quantity of the area is always positive. In order to transform this into a linear objective function, *S_i_* is segmented by the frontiers which are currently being evaluated. This creates regions labeled in Figure 2 as *r* in which the objective function remains constant. Each set of frontier end points is tested according to equation (5):

(5) $$\phi =\left(F_{j_{x_{1}}}^{i}F_{j_{y_{2}}}^{i}+a\prime_{i_{x}}F_{j_{y_{1}}}^{i}+a\prime_{i_{y}}F_{j_{x_{2}}}^{i}\right)\;-\;\left(F_{j_{x_{2}}}^{i}F_{j_{y_{1}}}^{i}+a\prime_{i_{x}}F_{j_{y_{2}}}^{i}+a\prime_{i_{y}}F_{j_{x_{1}}}^{i}\right)$$

ϕ=Vertex ordering test value

in which the signed quantity for area is determined and equation (6) to ensure a positive value for area.

(6) $$\begin{array}{ll} \beta_{j}^{i}\left(\Delta \right)= & \frac{1}{2}\;\left[F_{j_{x_{1}}}^{i}F_{j_{y_{2}}}^{i}-F_{j_{x_{2}}}^{i}F_{j_{y_{1}}}^{i}+a_{i_{x}}^{\prime }\;\left(F_{j_{y_{1}}}^{i}-F_{j_{y_{2}}}^{i}\right)+a_{i_{y}}^{\prime }\;\left(F_{j_{x_{2}}}^{i}-F_{j_{x_{1}}}^{i}\right)\right]|\phi >0\\ \beta_{j}^{i}\left(\Delta \right)= & \frac{1}{2}\;\left[F_{j_{x_{2}}}^{i}F_{j_{y_{1}}}^{i}-F_{j_{x_{1}}}^{i}F_{j_{y_{2}}}^{i}+a_{i_{x}}^{\prime }\;\left(F_{j_{y_{2}}}^{i}-F_{j_{y_{1}}}^{i}\right)+a_{i_{y}}^{\prime }\;\left(F_{j_{x_{1}}}^{i}-F_{j_{x_{2}}}^{i}\right)\right]|\phi <0. \end{array}$$

Since the only independent variables present are $a_{x}^{\prime }$ and $a_{y}^{\prime }$, it follows that the linear objective function for each frontier, *O_f_* should be given by equation (7):

(7) $$\begin{array}{ll} O_{f}^{i} & =\sum_{j}a_{i_{x}}^{\prime }\;\left(F_{j_{y_{1}}}^{i}-F_{j_{y_{2}}}^{i}\right)\;+a_{i_{y}}^{\prime }\;\left(F_{j_{x_{2}}}^{i}-F_{j_{x_{1}}}^{i}\right)\;|\phi >0\\ O_{f}^{i} & =\sum_{j}a_{i_{x}}^{\prime }\;\left(F_{j_{y_{2}}}^{i}-F_{j_{y_{1}}}^{i}\right)\;+a_{i_{y}}^{\prime }\;\left(F_{j_{x_{1}}}^{i}-F_{j_{x_{2}}}^{i}\right)\;|\phi <0, \end{array}$$

$$\begin{array}{l} O=\text{Linear objective function}\\ f=\text{Set of all frontiers} \end{array}$$

which is the linear objective function for each area formed by a frontier. The negative sum of these objective functions represents the maximization of the total area contained by all of the regions formed by agent’s position and any frontiers which are considered. For the minimization problem, the presence of sliver triangles along the boundaries formed by the frontiers traps the linear program in a local minimum at the agent’s position. However, the maximum formulation, when bounded, produces next best view estimations which allow the agents to discover new territory, and may utilize general guard locations rather than solely relying on vertex deployment.

#### 4.2.2 Bounding Set

In order for the feature-based information to be used in the heuristic for next best view calculation, a set of linear bounds must be established based on the lines forming the visible polygon, *S_i_*, for each agent, *a_i_*. These boundaries will be formed based on two cases, agent movement and agent deployment.

The requirements for successful deployment include the establishment of a visibly connected tree. This can be guaranteed for the process of moving one agent from its current position to a new position by exploiting the properties of star shaped polygons to bound the calculation of the next best view location problem. This work asserts that the visible region for each agent, is a star convex since there exists at least one point from which the entirety of *S_i_* may be viewed, $\left[a_{x_{i}},a_{y_{i}}\right]$ (Obermeyer et al., 2011). This point or region is referred to as the kernel of the star convex and is formed by the intersection of all interior half planes of the star-shaped polygon which is approximated using an algorithm depicted in Algorithm 3.

**Algorithm 3:** Define kernel.

**Input:** Load array of wall end points for agent *i* as *w*
**for** *All w* **do**
Calculate line $w_{x_{1}\;,y_{1}}{\bar{w}}_{x_{2},y_{2}}$;
Compare agent position *a_y_* to *w_y_* at *a_x_*;
**if** *a_y_* > *w_y_* **then**
*w* is a lower bound on the kernel *k_i_*;
**end**
**if** *a_y_* < *w_y_* **then**
*w* is an upper bound on the kernel *k_i_*;
**end**
**end**
**Input:** Load array of frontier end points for an agent *i* as *f*
**for** *All f* **do**
Calculate midpoint of each frontier *M*;
Define test points *T*_1_ = {*M_x_*, *M_y_* + 0.01} and *T*_2_ = {*M_x_*, *M_y_* –0.01};
**if** *T*_1_*is inside polygon* **then**
*f* is a lower bound on the kernel *k_i_*;
**end**
**if** *T*_2_*is inside polygon* **then**
*f* is an upper bound on the kernel *k_i_*;
**end**
**end**

We find the star kernel of the polygon formed by the environment features viewed by each agent which yields the bounded region in which the agent can move such that $\left[a_{x_{i}}^{\prime },a_{y_{i}}^{\prime }\right]\in S_{i}$ where $\left[a_{x_{i}}^{\prime },a_{y_{i}}^{\prime }\right]$ is the new position for agent *a*_i_. The definition of star convex states *S_i_* $S_{i}^{\prime }\subseteq S_{i}^{\prime }$ $S_{i}^{\prime }$, where *S_i_* is the visible region from the agent’s new position. The view location selection should be bounded such that $\left[a_{x_{i}}^{\prime },a_{y_{i}}^{\prime }\right]\in k_{i}$ where *k_i_* is the kernel of the star convex of the region visible to the *i*th agent.

### 4.3 Active Stationary

Active stationary agents are those for which the star kernel is a point. It is guaranteed that a star kernel exists for each agent in the simulation since the definition of the kernel is always satisfied by at least the robot’s current position. However, bounding a linear program by the agent’s current position fails to yield deployment locations or a movement location which would reveal more of the environment. To overcome this, the bounding functions must change. The active stationary agents act as static nodes in the connected tree from which new branches are formed.

#### 4.3.1 Objective Function

The objective function for an active mobile agent uses the same structure as the active mobile agents where the only independent variables present are *α_x_* and *α_y_*, and $O_{F_{j}}^{i}$ is calculated. This is the linear objective function for each area formed by a frontier. The sum of these positive objective functions results in the minimization of the area between the agent and each frontier. In order to allow for multiple agents to be deployed at a given time, it is efficient to cluster the frontiers into *q* groups and perform this optimization on each cluster yielding a set of agent deployment locations *a*_*i*=*i*+1,*i*+2,…*i+q*_ which describes the set of next best viewing points calculated from this stationary agent. This work uses a simplistic frontier clustering approach wherein frontiers are considered able to be grouped if every point, [*x*, *y*], in the region, *r_f_*, is contained such that, [*x*, *y*] ∈ *S_i_* holds where *S_i_* is the visible space of the stationary agent. This indicates that the region is a subset of the visible space. Using this strict clustering rule, only pairs of frontiers may be generated along with any remaining frontiers as singular clusters.

#### 4.3.2 Bounding Set

**Figure 3:**
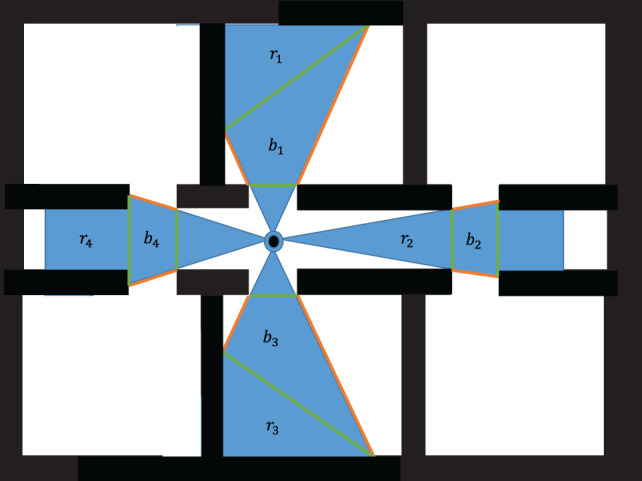
Frontier set bounding zones.

The bounding equations for active stationary agents are developed by collecting frontier pairs for which there exists some region *b* in which lines may be cast between the two frontiers which do not pass through any wall in the environment, i.e., the frontiers are visible to one another. For this work, the frontier end points were the only points at which this condition was verified. Therefore, frontiers which are only partially observable were not paired. This region, *b_i_*, is a subset of the region for which the equation for area is constant in terms of vertex ordering shown in Figure 3. The consistent vertex order ensures the objective function remains constant across the entire evaluated area.

Single frontiers are subject to an equality constraint which bounds the solution to the frontier itself. Therefore, an agent deployed to a cluster of frontiers with size 1 is deployed onto the frontier. Visual connection is maintained as long as the agents are deployed inside the star convex of an active agent, and each active agent is restricted to moving within the kernel of their star convex.

### 4.4 Inactive Stationary

**Figure 4:**
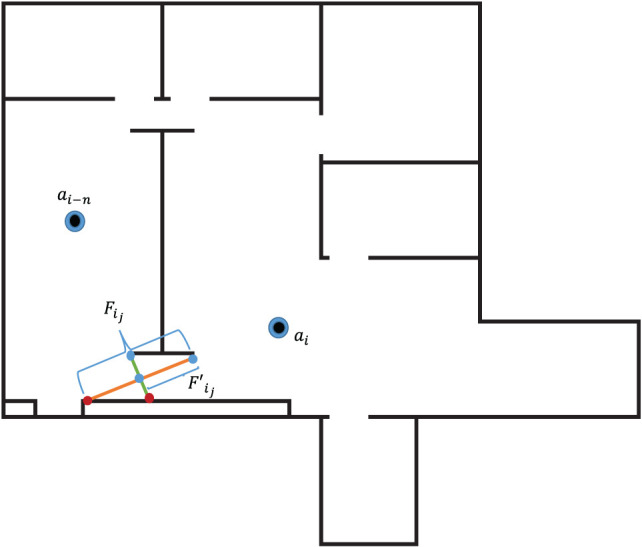
Frontier combination.

Agents pass their local maps and position data *via* the line of sight communication network at each step of the algorithm. Other robot’s maps are compared with the current automata’s visible space. In order to explore the environment without backtracking, each agent must avoid deploying new automata or moving to locations which have already been explored. This algorithm seeks to prevent backtracking through the examination of the frontiers which represent the boundaries for each agent’s visible region inside the environment. The frontier end points for each frontier visible to the current agent are tested against all other robots’ visible regions. If the end point lies inside a visible region, the frontier is redefined to reflect the boundary between discovered and undiscovered territory. The new frontier, $F_{i_{j}}^{2}$ lies between the end point of the previous frontier, $F_{i_{j}}$, and the intersection of the frontier visible by the previously deployed agent shown in Figure 4, *a*_*i*–*n*_.

An agent is considered IS if all frontier end points for that agent lie inside the visible space of any other deployed robot. When agents are considered IS, the program terminates.

## 5 Results and Discussion

**Figure 5:**
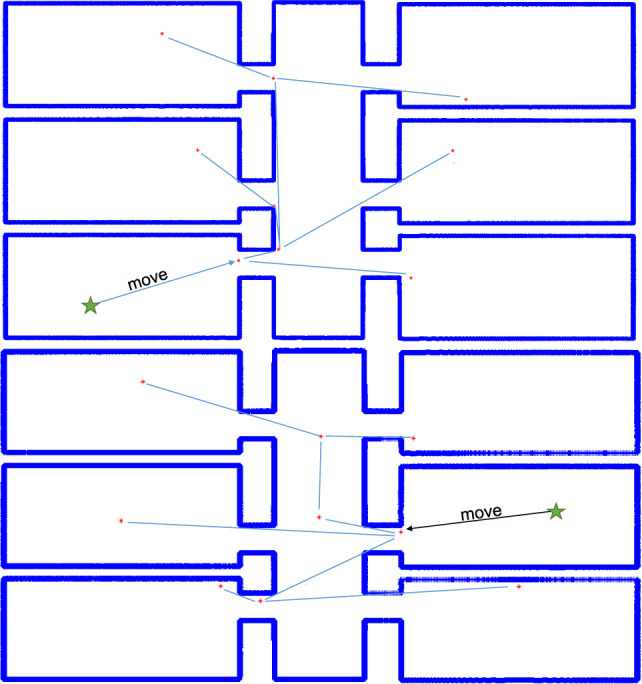
Simple rooms solution from 2 starting points.

Experiments were run utilizing MATLAB as a testing platform. Images of maps were supplied to MATLAB as portable network graphics (PNG) files and read into an occupancy grid based on the pixel count in the image. For the purposes of experimentation, an arbitrary scaling factor was chosen to reflect the true size of the room in meters. Two images were used for testing, “simple_rooms.png” and “autolab.png” from the set of stock images for the player project stage program (Mehrotra, 1992; Vaughan and Gerkey, 2007). The algorithm was run for “simple_rooms.png” from a set of randomly selected starting positions within the empty space. The solution for one such run is depicted in Figure 5 wherein the room was completely covered with 9 agents.

The number of agents sufficient to completely explore a polygonal environment with no holes, such as “simple_rooms.png,” is always equal to or less than *n*/3 where *n* is the number of vertices. In the case of “simple_rooms.png” the total number of vertices is 52 due to the thickness of each wall which indicates that 18 agents are always sufficient to cover the space. This sets a baseline “worst case” scenario for intelligent deployment of agents using the 3-coloring method to determine vertex guard placement. Full summary statistics of the trials presented in Table 1 which reveal a mean number of 10 agents covering the space with a maximum value 27% under worst-case.

**Table 1:** Comparison results.

		Algorithm				Deploy randomly along frontiers	
		Agents	Calculation time	Deployment steps	% agent reduction	Deployment steps	Agents
Simple rooms	Max	13.00	0.18	6.00	83.75	63.00	80.00
	Min	8.00	0.01	3.00	33.33	3.00	12.00
	Mean	10.34	0.05	4.36	41.33	5.76	17.63
	Median	10.50	0.05	4.00	30.00	5.00	15.00
Auto lab	Max	15.00	0.48	8.00	16.67	29.00	18.00
	Min	9.00	0.00	2.00	25.00	3.00	12.00
	Mean	11.78	0.06	4.72	18.08	5.16	14.38
	Median	12.00	0.04	5.00	14.29	4.00	14.00

**Figure 6:**
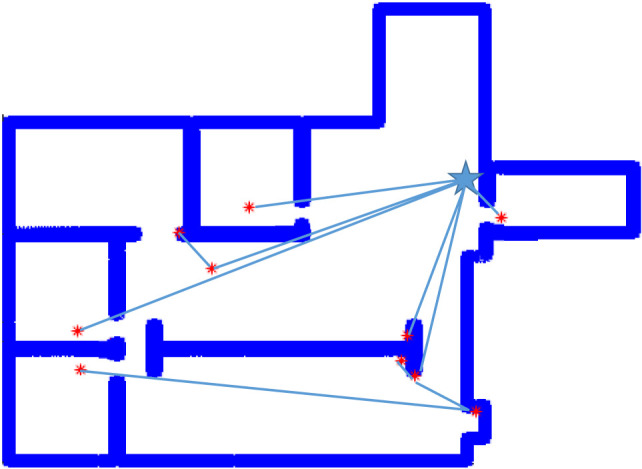
Autolab solution.

The number of deployment steps is the number of individual robot requests for additional agents needed before the algorithm concluded. These values are much closer, but the algorithm still outperforms random placement by 24%. These values are expected to be closer together due to agent behavior. The number of agents deployed by the random frontier exploration at each deployment step is equal to the number of exploration boundaries visible to an agent which are not in another agent’s field of view. Therefore, the number of agents deployed should be much larger, while exploration speed improves due to the sheer number of robots being deployed. Both the proposed linear program and random deployment suffer due to wall thickness. The star-convex-based deployment techniques ensure visual connectivity, but if an agent is in doorway or close to a wall, the bounding region for deployment becomes very small, thus limiting the utility gain for that deployment cycle. Allowing an agent to reposition improves this, but does not entirely offset that limitation of the star polygon method. In the case of “autolab.png” the proposed solution also ran to a state of complete coverage. A selected iteration from one starting location provided complete coverage with a set of 11 agents illustrated in Figure 6.

Holes in the environment, such as the wall segment in the center of “autolab.png” present a number of challenges to an agent exploring the space. One prominent issue is that of agent overlap. This was definitely an issue regarding deployment with the proposed algorithm. One limitation set by the chosen solution was the inability to deal with pseudo-wall clusters of more than 2. The hole provided situations in which multiple exploration frontiers may have been serviced by a conservative single-agent deployment, but the pairing limitation prevented this from occurring. Exploration frontier pairing did, however, allow for the agents to completely explore the environment with an 18% reduction in total robots used over randomly deploying along frontiers. The issue of number of agents deployed resulting in faster coverage time was exacerbated by the hole in the environment. It more than halved the improvement in average number of deployment cycles from 24.3% in “Simple_rooms.png” to 9% in “autolab.png.” This reduction is likely due to the breadth-first deployment of agents in the new method causing a number of low-utility deployments first followed by higher value deployments in strings later in the execution. As each wave of agents is requested, their ID numbers increment. In “Simple_rooms.png,” The first agent either deployed agents to each doorway if it was randomly started in the hallway, or deployed a single agent into the hallway if it was initialized in a room. This means that the ID number of the highest utility agent was either 1 or 2. Since agents evaluate and deploy sequentially, this means that the highest value agent usually was allowed to request robots first. In the case of “autolab.png” the complexity of the environment meant that agents were assigned to many frontier pairs from any starting agent’s position. Only by happenstance, then, did the highest utility agents receive ID numbers low enough to ensure efficient deployments following the initial spread. In both cases, the algorithm only requested that the initial agent deployed in the environment move. No other agent repositioning was observed as the optimization for active and immobile agents always deployed new robots to location for which the new agents’ star-kernels were points, or the agents were immediately inactive stationary due to a lack of unexplored frontiers. This means that, aside from the first agent, all deployments were one-shot with no need to reposition.

## 6 Conclusion

The presented algorithm provides a decentralized solution to the problem of determining the next best observation point for each agent in a team of autonomous robots engaged in exploring a previously unknown environment. Each automata seeks to maximize the area revealed by their next action through observation of the geometric features in the agent’s observable space as well as the discovered area transmitted *via* line-of-sight communication to the currently acting robot. The proposed algorithm was able to ensure complete coverage of both a simple polygonal environment and a complex environment with a hole while reducing the number of agents used y an average of 41 and 18%, respectively, over randomly deploying agents along the exploration boundaries. Even though agent count was significantly reduced, the total number of deployment cycles and robot movements was kept to an average of 4.36 for the simple and 4.71 for the complex environment. This translates to a 24.3% decrease in deployment cycles over random deployment to each available frontier for simple environments and 9% reduction for the complex environment. It should be noted that the limitations of this work are significant as only simulation was performed to validate the performance of the algorithm, and specific environmental factors such as size, shape, and number of holes were not addressed. The algorithm appears to be applicable to any static polygonal environment in which it is possible to collect both localization data and a detailed scan of the walls and features of the space. That said, the proposed algorithm successfully reduced the number of agents used in comparison to random deployment without relying on any agent repositioning other than the first robot deployed. Since the calculations of next-best-view only took a maximum of 0.48 s to complete, the primary time sink in the deployment process would be agent movement. The one-shot deployment observed in the execution of the proposed solution has the potential to significantly reduce total deployment time while also reducing the total number of robots required to complete the exploration.

## Future Work

This algorithm could be improved through the inclusion of a more comprehensive strategy for field-of-view overlap prevention which could both reduce superfluous agent deployment and improve algorithm termination accuracy. Additionally, agent deployment order needs to be addressed such that agents with a higher probability of discovery or a maximal utility are serviced before agents of lower value. Furthermore, more simulation is required in order to characterize the algorithm based on number of holes in the environment as “autolab.png” only contains one hole, number of walls, average width of hallways, etc. Following this study, a major step for future work is to move the strategy from pure simulation onto a physical robot platform for testing. This will require an extension of the deployment constraints to cope with sparse or noisy laser scan data which has been shown to produce false pseudo walls.

## Author Contributions

SC contributed to problem identification, high-level solution development, and general guidance. RG contributed to developing those ideas, modifying them, debugging, extensive software implementation, and verification.

## Conflict of Interest Statement

The authors declare that the research was conducted in the absence of any commercial or financial relationships that could be construed as a potential conflict of interest.

## References

[B1] Aguilera F. Urdiales C. Sandoval F. (2015). “An evaluation of two distributed deployment algorithms for mobile wireless sensor networks,” in Ubiquitous Computing and Ambient Intelligence. Sensing, Processing, and Using Environmental Information. Lecture Notes in Computer Science, Vol. 9454, eds García-Chamizo J. Fortino G. Ochoa S. (Cham: Springer).

[B2] Andre T. Bettstetter C. (2016). Collaboration in multi-robot exploration: to meet or not to meet? J. Intell. Robot. Syst. 82, 325 10.1007/s10846-015-0277-0

[B3] Bautin A. Simonin O. Charpillet F. (2012). “MinPos: a novel frontier allocation algorithm for multi-robot exploration,” in Intelligent Robotics and Applications. ICIRA 2012. Lecture Notes in Computer Science, Vol. 7507, eds Su C. Y. Rakheja S. Liu H. (Berlin, Heidelberg: Springer).

[B4] Boxer L. Chang C.-S. Miller R. Rau-Chaplin A. (1993). Polygonal approximation by boundary reduction. Pattern Recognit. Lett. 14, 111–119. 10.1016/0167-8655(93)90084-Q

[B5] Cepeda J. S. Chaimowicz L. Soto R. Gordillo J. L. Alanís-Reyes E. A. Carrillo-Arce L. C. (2012). A behavior-based strategy for single and multi-robot autonomous exploration. Sensors 12, 12772–12797. 10.3390/s120912772

[B6] Chvatal V. (1975). A combinatorial theorem in plane geometry. J. Comb. Theory B 18, 39–41. 10.1016/0095-8956(75)90061-1

[B7] Cortés J. (2008). “Area-constrained coverage optimization by robotic sensor networks,” in Decision and Control, 2008. CDC 2008. 47th IEEE Conference on (Cancun: IEEE), 1018–1023.

[B8] Costanzo C. Loscrí V. Natalizio E. Razafindralambo T. (2012). Nodes self-deployment for coverage maximization in mobile robot networks using an evolving neural network. Comput. Commun. 35, 1047–1055. 10.1016/j.comcom.2011.09.004

[B9] Fernandes L. A. Oliveira M. M. (2008). Real-time line detection through an improved hough transform voting scheme. Pattern Recognit. 41, 299–314. 10.1016/j.patcog.2008.04.007

[B10] Ganguli A. Cortés J. Bullo F. (2006). “Distributed deployment of asynchronous guards in art galleries,” in American Control Conference, 2006 (Minneapolis, MN: IEEE), 6.

[B11] Ganguli A. Cortés J. Bullo F. (2007). “Visibility-based multi-agent deployment in orthogonal environments,” in American Control Conference, 2007. ACC’07 (New York, NY: IEEE), 3426–3431.

[B12] González-Banos H. H. Latombe J.-C. (2002). Navigation strategies for exploring indoor environments. Int. J. Robot. Res. 21, 829–848. 10.1177/0278364902021010834

[B13] Heckbert P. S. Garland M. (1997). Survey of Polygonal Surface Simplification Algorithms. Technical Report. Fort Belvoir, VA: Carnegie-Mellon Univ Pittsburgh PA School of Computer Science.

[B14] Howard A. Matarić M. J. Sukhatme G. S. (2002). An incremental self-deployment algorithm for mobile sensor networks. Auton. Robots 13, 113–126. 10.1023/A:1019625207705

[B15] Kröller A. Baumgartner T. Fekete S. P. Schmidt C. (2012). Exact solutions and bounds for general art gallery problems. J. Exp. Algorithmics 17, 2–3.

[B16] Mehrotra S. (1992). On the implementation of a primal-dual interior point method. SIAM J. Optim. 2, 575–601. 10.1137/0802028

[B17] Nguyen V. Martinelli A. Tomatis N. Siegwart R. (2005). “A comparison of line extraction algorithms using 2d laser rangefinder for indoor mobile robotics,” in Intelligent Robots and Systems, 2005.(IROS 2005). 2005 IEEE/RSJ International Conference on (Edmonton: IEEE), 1929–1934.

[B18] Obermeyer K. J. Ganguli A. Bullo F. (2011). Multi-agent deployment for visibility coverage in polygonal environments with holes. Int. J. Robust Nonlinear Control 21, 1467–1492. 10.1002/rnc.1700

[B19] O’rourke J. (1987). Art Gallery Theorems and Algorithms, Vol. 57 Oxford: Oxford University Press.

[B20] Potthast C. Sukhatme G. S. (2014). A probabilistic framework for next best view estimation in a cluttered environment. J. Vis. Commun. Image Represent. 25, 148–164. 10.1016/j.jvcir.2013.07.006

[B21] Schwager M. Rus D. Slotine J.-J. (2011). Unifying geometric, probabilistic, and potential field approaches to multi-robot deployment. Int. J. Robot. Res. 30, 371–383. 10.1177/0278364910383444

[B22] Solanas A. Garcia M. A. (2004). “Coordinated multi-robot exploration through unsupervised clustering of unknown space,” in Intelligent Robots and Systems, 2004.(IROS 2004). Proceedings. 2004 IEEE/RSJ International Conference on, Vol. 1 (Sendai: IEEE), 717–721.

[B23] Stachniss C. (2009). Robotic Mapping and Exploration, Springer Tracts in Advanced Robotics, Vol. 55, eds Siciliano B. Khatib O. Groen F. (Berlin, Heidelberg: Springer-Verlag).

[B24] Vaughan R. T. Gerkey B. P. (2007). “Reusable robot software and the player/stage project,” in Software Engineering for Experimental Robotics. Springer Tracts in Advanced Robotics, Vol. 30, ed. Brugali D. (Berlin, Heidelberg: Springer).

[B25] Zhang Y. (1998). Solving large-scale linear programs by interior-point methods under the MATLAB environment. Optim. Methods Software 10, 1–31. 10.1080/10556789808805699

